# ABCDscores: an R package for computing summary scores in the ABCD Study®

**DOI:** 10.3389/fninf.2026.1858950

**Published:** 2026-08-14

**Authors:** Le Zhang, Olivier Celhay, Biplabendu Das, Samantha Berman, Laura R. Ziemer, Calen J. Smith, Shermaine Abad, Anders M. Dale, Janosch Linkersdörfer

**Affiliations:** Center for Multimodal Imaging and Genetics, J. Craig Venter Institute, La Jolla, CA, United States

**Keywords:** ABCD study, longitudinal study, neuroinformatics, R package, summary scores

## Abstract

The ABCD Study® is the largest longitudinal research study of brain development and child health across the United States. The study generates a rich multimodal dataset, including file-based raw and derivative data and a curated tabulated dataset comprising data from a broad spectrum of research domains. Historically, summary scores in the tabulated dataset were computed using the electronic data capture system REDCap or external scripting solutions. However, this approach was rigid, unreliable, and lacked transparency around how scores were computed. Here, we introduce ABCDscores, an R software package that is used by the study to compute most non-proprietary summary scores included in the tabulated ABCD data resource and allows for customization to meet specific research needs. ABCDscores provides a flexible, open-source solution that uses R for transparent and reproducible scoring. The package is built to apply standard scoring methods across different types of assessments, ensuring consistent and accurate results even with large datasets. By simplifying complex scoring procedures into a user-friendly process, ABCDscores streamlines research workflows, reduces errors, and saves researchers valuable time. The package aims to improve analyses of data from the ABCD Study® and can serve as a model for the development of similar tools and a standardized framework for other research studies, fostering new discoveries and innovation in the field.

## Introduction

The Adolescent Brain Cognitive Development (ABCD) Study is the largest long-term study of brain development and child health in the United States, following more than 11,000 youth from ages 9–10 into early adulthood. This comprehensive, longitudinal cohort study collects a vast array of data, including neuroimaging, genetics, neurocognitive assessments, behavioral surveys, biospecimens, wearables, and environmental measures, across 21 research sites nationwide (Feldstein Ewing and Luciana, 2018). Large-scale research initiatives, such as the UK Biobank and the Human Connectome Project (Sudlow et al., 2015; Van Essen et al., 2013), have established benchmarks in open science by offering comprehensive, de-identified datasets to the international scientific community, thereby expediting advancements in the field. Following this open-science model, the ABCD Study® provides its extensive, longitudinal datasets to researchers globally and promotes diverse investigations into adolescent development. The ABCD Study’s data are publicly available through the NIH Brain Development Cohorts (NBDC) Data Hub (ABCD Consortium, 2026).

A critical aspect of preparing ABCD data releases involves the computation of summary scores. These are composite measures derived from various assessments within the tabulated dataset. Summary scores are essential for capturing complex constructs such as cognitive functioning, mental health status, or substance use. Calculating these scores requires meticulous data processing, including handling missing data, applying appropriate scoring algorithms, and ensuring consistency across different data releases.

Originally, the ABCD Study® employed an inconsistent approach to compute summary scores. Some scores were generated as calculated fields within the Research Electronic Data Capture (REDCap) system used for data acquisition, while others were computed through external workflows utilizing various programming languages and methods. Despite its widespread acceptance in clinical research (Patridge and Bardyn, 2018; Harris et al., 2009), a notable limitation of REDCap is its relatively rigid structure, which can hinder the implementation of complex algorithms. Although REDCap serves as a valuable tool for data collection and management, its built-in functionalities often fall short when researchers need to integrate or apply sophisticated computational models (Ndlovu et al., 2024). Consequently, previous solutions for computing summary scores in ABCD required the inclusion of external workflows, which, over time, resulted in inconsistent methodologies and fragmented scripts across different programming languages.

Additionally, the summary scores included in prior ABCD releases exhibited a lack of transparency and reproducibility, as the scoring algorithms were not publicly accessible and were inadequately documented. This made it challenging for data users to reproduce the scores without soliciting disclosure from the ABCD Consortium. Even if they managed to acquire the scripts, the algorithms varied between releases. In contrast, typical extensions, or packages, in languages such as R and Python are distributed as versioned releases, allowing users to download the source code from version-control platforms such as GitHub. The previous ABCD summary score scripts were poorly organized and lacked systematic version control. Together, these limitations posed significant challenges for developers aiming to extend existing algorithms and incorporate new measurements, as well as for researchers striving to reproduce the scores.

To address these challenges and streamline the research process, we introduce ABCDscores, an R package designed to automate the computation of most non-proprietary summary scores published by the ABCD Study®. In this manuscript, we detail the development and functionalities of the ABCDscores R package. We provide an overview of the included summary scores, the modular utility functions we use to compute the summaries, and discuss the implementation of standardized scoring procedures. We further provide use cases to demonstrate the package’s utility. ABCDscores aims to enhance reproducibility, allow flexibility, reduce errors, and save valuable time for researchers by encapsulating the scoring algorithms and data processing steps into a user-friendly, open-source package. We hope this manuscript will contribute to existing and future research, provide a roadmap for standardized summary score computations, and foster further innovation in this critical area.

## Methods and implementation

ABCDscores was developed as an R package. The package is designed to automate the computation of standardized summary scores from the ABCD Study® datasets. It allows users to reproduce the summary scores that are being released as part of the ABCD tabulated data resource or to produce customized scores.

### Coding style

ABCDscores adheres to the Tidyverse style guide for writing R code, ensuring consistency, readability, and ease of use (Wickham et al., 2019). By following this guide, we ensure that our code is not only efficient but also easily understandable, facilitating collaboration and future development.

### Core design

The package architecture follows modular design principles, separating core functionality into distinct components to improve clarity, flexibility, and maintainability. Each summary score algorithm is implemented as a standalone function with consistent input/output patterns, making it easy for researchers to inspect the logic for a given score and incorporate specific measures into their workflows.

All scores are organized by their ABCD research domain. Currently, scores are available for the following seven domains: ABCD General (*ab*), Family, Friends & Community (*fc*), Mental Health (*mh*), Neurocognition (*nc*), Novel Technologies (*nt*), Physical Health (*ph*), and Substance Use (*su*). Within each domain, scores are organized by database table. Each table contains data for a specific survey, questionnaire, or measure administered to either the youth participant or their parent. Within each table, scores may be grouped into scales and/or subscales. For instance, the Family Environment Scale [Parent] and [Youth] tables, (*fc_p_fes*) and (*fc_y_fes*), respectively, are part of the *fc* domain. These scales include subscales like the *cohesion (cohes)* and *conflict (confl)* subscales, resulting in scores like *fc_p_fes__cohes_mean* (mean of items in the parent cohesion subscale) and *fc_p_fes__confl_mean* (mean of items in the parent conflict subscale). Each scale/subscale can have one or several summary scores. For example, most means and sums are accompanied by *_nm* (number missing) scores, which provide the number of items that were missing for the computation of a specific score (e.g., *fc_p_fes__cohes_nm*). In the source code, scores are organized by domain, with one R file per domain which are named with the prefix “*scores_*” (e.g., “scores_ab.R,” “scores_fc.R,” *etc*.). There are a few exceptions: the “scores_mh_aseba.R” (ASEBA, Achenbach System of Empirically Based Assessment), “scores_su_tlfb_sui_sdsu.R” (Substance Use), “scores_mh_famhx.R” (Family History), “scores_ph_meds.R” (Medication), and “scores_nt_fitbit.R” (Fitbit) files, which are separated into their own files due to their complexity and size.

Score functions in the package are designed in a consistent way (Supplementary Figure S1):

1. Function naming: All functions that compute summary scores begin with the prefix “*compute_*,” followed by the specific score name in the ABCD tabulated dataset. For example, “*compute_mh_p_ders__attun_mean*” indicates that it computes the summary score “*mh_p_ders__attun_mean*,” the mean of the DERS (Difficulties in Emotion Regulation Scale) attunement subscale.
2. Input: The function expects a data frame containing the variables that are needed for the score calculation. This data frame can be a subset of the larger ABCD dataset, allowing users to focus on specific participants or measurements.
3. Structure of the function: (a) *Validates* the inputs to all parameters, including but not limited to checking if the data frame contains the required variables, if the variables are of the correct type, and if other parameters are within the expected range. If the parameters do not pass validation, the function will return an informative error message to the user. (b) *Computes* the score using the scoring algorithm, and finally, (c) *Returns* the computed score in a desired format.
4. Output: The function returns a data frame containing the computed summary scores, attached to the original data frame (default) or as a separate data frame, depending on the user’s preference.

Functions with the *_all* suffix are high-level convenience functions designed to compute all summary scores within a specific scale/measure at once. For example, “*compute_ph_y_bp_all*” computes all summary scores within the Youth Blood Pressure measure, which includes 6 summary scores: *ph_y_bp__sys_mean*, *ph_y_bp__sys_nm*, *ph_y_bp__dia_mean*, *ph_y_bp__dia_nm*, *ph_y_bp__hrate_mean*, and *ph_y_bp__hrate_nm* (systolic, diastolic, and heart rate, respectively).

### Scoring algorithm and utility functions

The scoring algorithms were developed in close collaboration with the ABCD Study® domain experts and assessment workgroups. The experts specified the scoring algorithms, handling of missing data, and any specific transformations required. The development team translated these specifications into R code, generated both simulated and real results for the experts to review, and iteratively refined the code based on their feedback. This collaborative approach ensured that the scoring algorithms accurately reflected the intended measures.

To enhance code readability and maintainability, we developed a set of utility functions that are used across multiple scoring algorithms. These utility functions consist of common operations, such as computing means, sums, and the number of missing values, as well as more complex utility functions which, for example, perform row-wise counts based on user-defined conditions.

ABCDscores utility functions are designed to be reusable and modular, allowing for easy integration into different scoring algorithms. In ABCDscores, all score-related utility functions are prefixed with “*ss_*” (e.g., *ss_mean*, *ss_sum*, *ss_nm, ss_count*). This naming convention clearly indicates their purpose and distinguishes them from other functions in the package. In addition to the summarizing utility functions, ABCDscores contains several utility functions used for data transformations, which provide wrapper functions around powerful tidyverse functions, such as combining two columns into one (*combine_cols*), recoding values in a column (*recode_levels*), and converting a longitudinal variable into a static one (*make_static*). Together, these generic utility functions in ABCDscores can be used to create other summary scores within and beyond ABCD data.

### Documentation

The ABCDscores package is well documented using *roxygen2* comments for function documentation and vignettes providing details about how to use the package. The documentation is also provided as a static website that is created using the *pkgdown* package: https://software.nbdc-datahub.org/ABCDscores/. As part of the function documentation for each score, we provide detailed information about the scoring algorithm, including the rationale behind the scores, and the citations for validated measures that the scores were originally derived from. For scales with more complicated input data requirements and scoring algorithms, e.g., the ASEBA and SUI/TLFB scores, we provide detailed vignettes to guide users through preparing the input data and computing the scores.

### Defaults and customization

The ABCDscores package is designed to be user-friendly and flexible, allowing users to customize the scoring process according to their specific needs. The package provides default arguments for each scoring function, which are set to recommended values based on experts’ guidelines. These defaults ensure that users can reproduce the exact scores that are published as part of the ABCD tabulated data resource. However, to enable researchers to create scores according to their specific requirements or preferences, we allow users to change these default arguments to suit their specific needs.

All scoring functions in the package have a common set of standard arguments:

- Data: The input data frame containing the relevant variables for the score calculation. This is a required argument.
- Name: The name of the score column in the output data frame. The default is always the function name without the “*compute_*” prefix, i.e., the name of the respective score in the ABCD tabulated dataset.
- Combine: A logical argument indicating whether to combine the computed score with the original data frame. The default is *TRUE*, meaning that the score will be added to the original data frame.

Depending on the specific scoring function, there may be additional default arguments that are specific to the domain or scale, for example, the maximum number of missing items allowed while still computing the score. These arguments are described in the function documentation and can be customized as needed.

## Results

### Overview of ABCDscores

**Figure 1:**
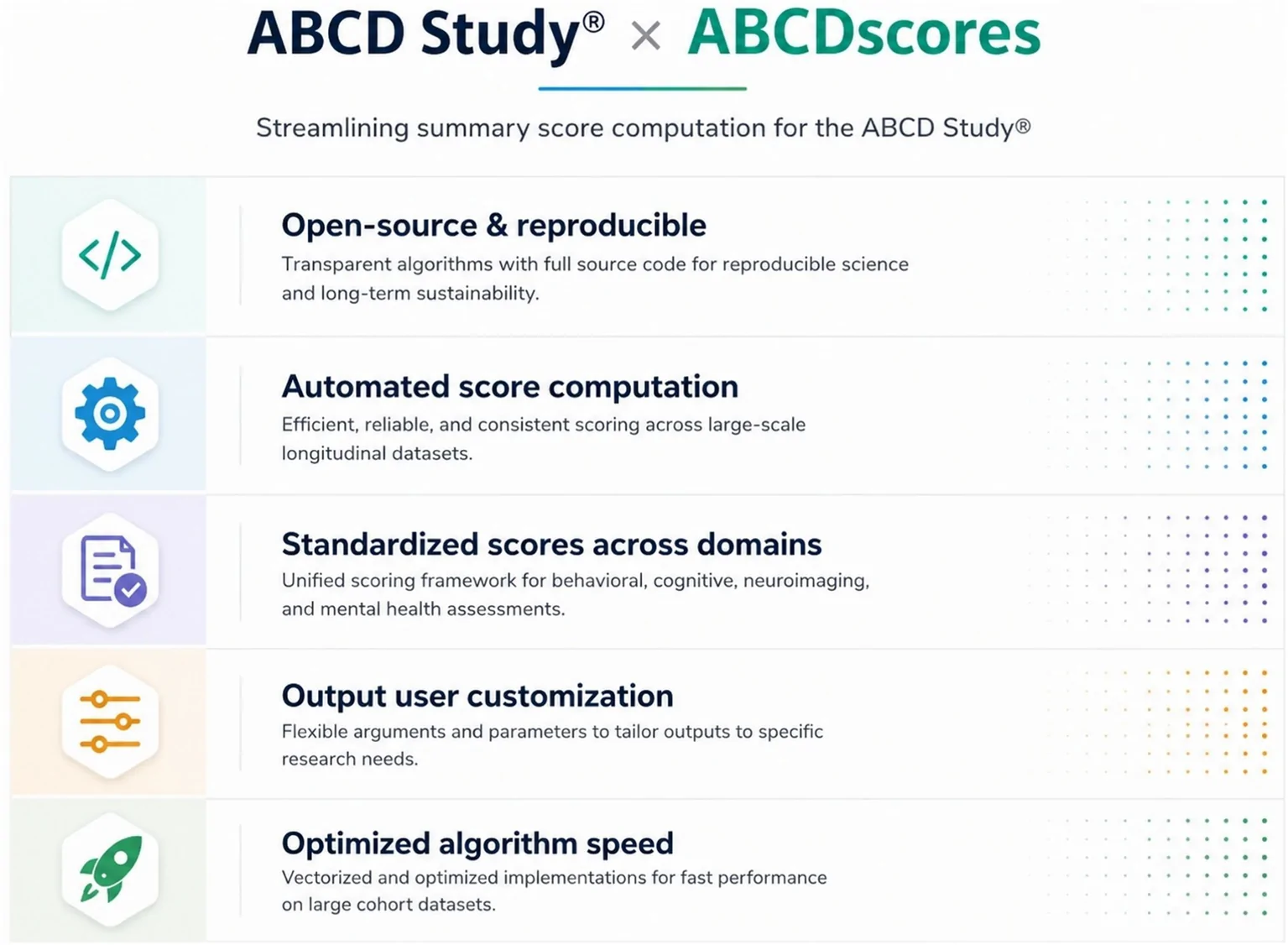
Overview of ABCDscores features in support of the ABCD Study®. This figure highlights the core functionalities of the ABCDscores R package.

As an open-source R package designed to enhance data processing transparency and reproducibility for the ABCD Study® (Figure 1), ABCDscores calculates summary scores across multiple domains, such as behavioral, cognitive, and mental health (Table 1). A total of 889 summary score functions and 17 utility functions are included in ABCDscores v7.0.0. In most cases, one score function corresponds to one summary score (one-to-one functions). However, a subset of functions follows a configuration-driven approach in which a single function can generate multiple summary scores (one-to-many functions), particularly in the Physical Health, Mental Health, and Substance Use domains. For example, the score functions for *su_y_sui* (Substance Use Interview [Youth]) and *su_y_tlfb* (Timeline Followback Interview Results [Youth]) can each be used to compute multiple summary scores. Rather than defining a separate function for every score, as is done elsewhere in the package, these functions are parameterized so that the same underlying computation can be applied across different substances and, for the TLFB functions, across additional dimensions such as recall period, weekday versus weekend use, co-use, and binge threshold. This modular design avoids creating near-duplicate functions for every substance and parameter combination while also allowing users to compute additional custom summary scores beyond those released in the ABCD data resource by varying the function parameters. As a result, the number of summary scores substantially exceeds the number of functions in these domains (Table 1). In total, 3,231 scores in the ABCD 7.0 data release were computed using ABCDscores. For future package releases, we expect more functions to be added, empowering numerous new summary scores to be calculated.

**Table 1:** Number of score functions included in the 7.0.0 release of the package and number of scores computed in the ABCD data release 7.0 by research domain.

Domain	Functions	Scores
Neurocognition	5	5
Novel technologies	10	10
ABCD general	11	11
Physical health	108	1,153
Family, friends and community	101	101
Mental health	546	671
Substance use	108	1,280
Utility functions	17	N/A
Total	906	3,231

Besides the main score functions, the ABCDscores package contains multiple generic, reusable utility functions that will enable users to compute their own summary scores within and beyond the ABCD Study®. A list of all generic utility functions available in the ABCDscores package is provided in Table 2. More details about each utility function can be found at: https://software.nbdc-datahub.org/ABCDscores/reference/index.html#utility-functions.

**Table 2:** Overview of utility functions provided in ABCDscores.

Generic utility functions	
Score computation	Data transformation
ss_sum	recode_levels
ss_mean	combine_cols
ss_nm	combine_levels
ss_count	combine_checkboxes
ss_count_cond	make_static
ss_max	
ss_prsum	
ss_mean_pos	
ss_tscore	
ss_sum_mh_ple	
ss_mean_mh_ple	
ss_nm_mh_ple	

### Use cases

**Figure 2:**
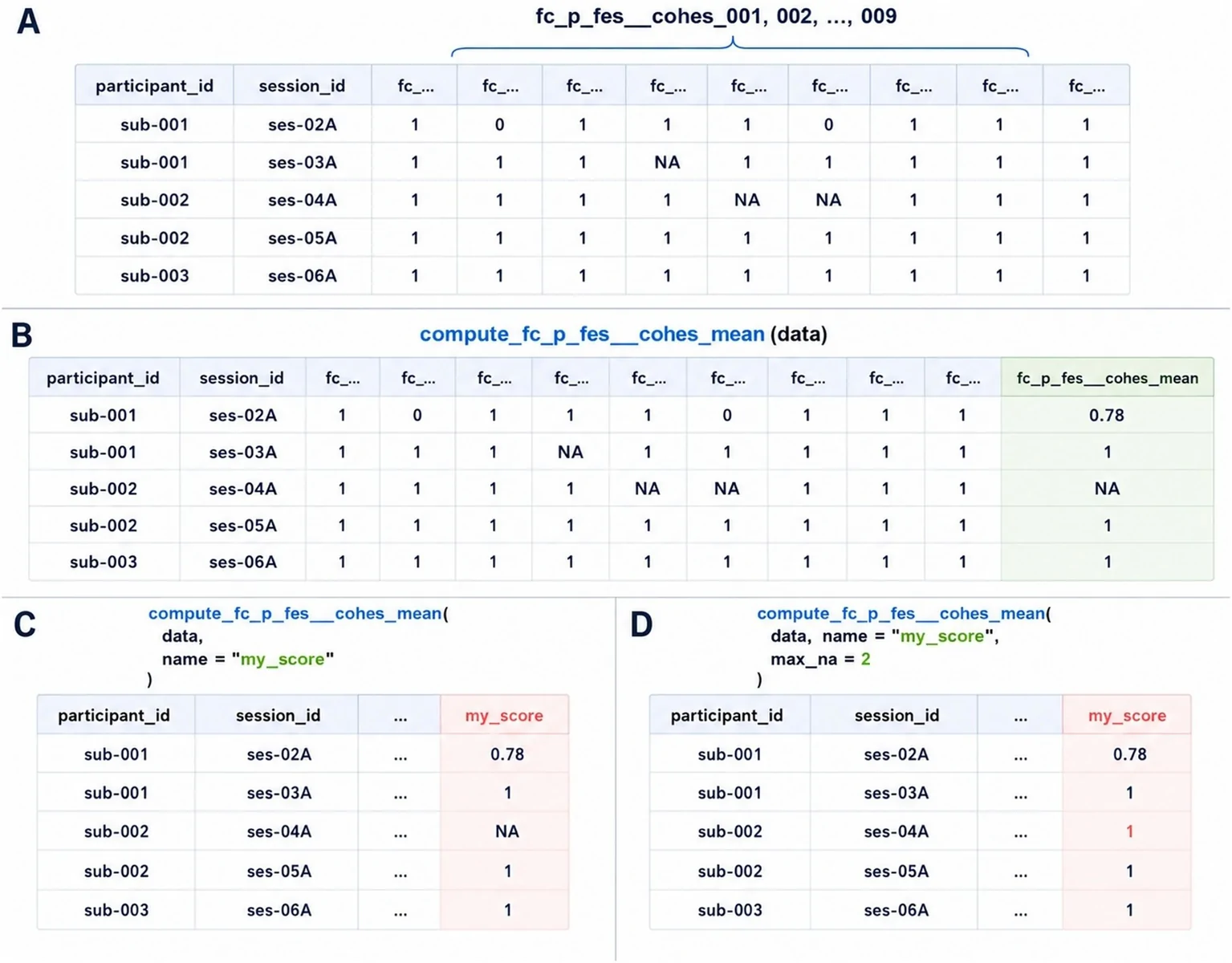
Use case examples of computing scores with the package using simulated data. **(A)** Simulated item-level input data of the family environment scale [parent] cohesion subscale, which contains 9 items (*fc*_*p*_*fes*__*cohes*_001, 002, …, 009). **(B)** Example of using all the default arguments to compute the *fc_p_fes__cohes_mean* score. **(C)** Customizing the output column name of the score. **(D)** Further customizing the maximum number of missing items allowed to 2 (default 1), so some previously *NA* row(s) are calculated with values.

The following examples showcase some basic behaviors and advanced customization of computing summary scores with ABCDscores. In these examples, we use some simulated data for the Family Environment Scale [Parent] cohesion subscale (*fc_p_fes__cohes*) to illustrate how most score functions behave and how to apply customizations when using ABCDscores (Figure 2).

1. To compute the *fc_p_fes__cohes_mean* score, the input data frame needs to contain all the required columns. In this case, they are the *fc_p_fes__cohes_001*, *002, …, 009* columns (Figure 2A).
2. When the user uses all the default arguments, the only required input for the *compute_fc_p_fes__cohes_mean* function is a data frame containing the required columns. The output score is appended to the end of the original input as a new column with name “*fc_p_fes__cohes_mean*” (Figure 2B).
3. As a simple customization, users can, for example, change the column name for the score in the output data frame (Figure 2C). In this example, the name was changed from “*fc_p_fes__cohes_mean*” to “*my_score*.”
4. In the previous example, the final results remained unchanged compared to (2). However, users can also decide to modify parameters that change how the function computes the score and result in different results. In this example, the *max_na* argument (maximum number of missingness allowed) was changed from 1 (default) to 2. As a result, a summary score for the third row is now calculated by the function (Figure 2D).

In addition to the *max_na* argument, there are several other parameters that can be used to adjust the stringency, range, or variability of scores. All of these parameters come with pre-defined defaults established by the ABCD Study assessment workgroups. Therefore, generating the scores from the required input data is straightforward, and these advanced parameters are intended for use by experts with particular research objectives.

## Discussion

### Performance and scalability

ABCDscores was developed with attention to both computational efficiency and scalability to large cohort datasets. Time-intensive steps in the scoring workflow were systematically identified during development and, where feasible, replaced with vectorized operations and reusable utility functions. These design choices improved runtime and reduced computational overhead without compromising code clarity or reproducibility. In a reproducible benchmark on the full ABCD 7.0 tabulated release, the benchmark-eligible summary scores in the 7.0 release (2,240 scores) were generated in roughly 6–7 min of single-threaded compute time, with individual scoring functions only taking a few tens of milliseconds each. Full benchmark details are provided in the package documentation (https://software.nbdc-datahub.org/ABCDscores/articles/benchmark.html). In practice, ABCDscores can efficiently process large ABCD Study® datasets without the need for multi-core parallelization or specialized hardware. This makes the package scalable to full data-release processing, including computation of all scores for releases, such as ABCD 7.0, while remaining accessible on a modern, everyday-use laptop. By combining efficient implementation with a modular architecture, ABCDscores supports reproducible score generation across both routine research use and larger standardized data-processing workflows.

### Limitations

ABCDscores primarily targets ABCD tabulated data, and the package assumes ABCD-compatible input structure and variable naming, which may limit direct portability to other ABCD or outside datasets without prior harmonization. Although optimized for routine use on modern laptops, performance may vary with memory constraints and preprocessing choices. Although the package is implemented in R to ensure statistical reliability and CRAN compliance, it can be integrated into Python-based workflows using tools like rpy2, allowing researchers to leverage these tools effectively regardless of their preferred programming language. As ABCD releases evolve, continued maintenance is required to preserve release-to-version compatibility.

## Conclusion

ABCDscores is an R package designed to automate the computation of summary scores for the ABCD Study®. By integrating complex scoring algorithms and data processing steps, ABCDscores streamlines analysis workflows, enhances reproducibility, and reduces human error. ABCDscores efficiently handles large datasets, providing accurate and consistent summary scores across various research domains. In conclusion, ABCDscores significantly enhances the analysis of ABCD Study® data, thereby allowing researchers to concentrate on scientific discovery and accelerating research into adolescent brain and cognitive development.

## Data Availability

The data analyzed in this study are available through the NBDC Data Hub, subject to an approved Data Use Certification at https://www.nbdc-datahub.org/data-access-process. This article describes the ABCDscores package and its functions for computing summary scores from the required ABCD input data. No participant-level data are included in the article.
