# Supplementary material for: ABCDscores: an R package for computing summary scores in the ABCD Study®
Source: Front Neuroinform. 2026 Aug 14;20:1858950. doi: 10.3389/fninf.2026.1858950 (PMC13522172; doi:10.3389/fninf.2026.1858950)
Supplement: Supplementary file 1 [file Data_Sheet_1.DOCX]

## Supplement for ABCDscores: An R package for computing summary scores in the ABCD Study®

## Figures

**Figure S1**. Architecture and workflow of the ABCDscores R package. (A). Overview of the modular structure of ABCDscores. Scores are organized by domain. Each domain (*e.g*., *ab* for ABCD General, *mh* for Mental Health, etc) includes dedicated functions prefixed with compute_<domain>_... to calculate domain-specific scores. (B). Within each domain, multiple summary score functions, such as sum, mean, and others, are available for each scale or measure. Each function corresponds to an individual score. Additionally, there is an “all” function, indicated by the *“_all”* suffix, which computes all scores within a given scale or measure simultaneously. (C). Scoring workflow: A dataframe containing the required raw score columns is passed to the compute functions, optionally including user-defined arguments. The output can either be the original dataframe with the newly appended score columns or a separate dataframe containing only the calculated scores, depending on the user’s preference.
